# Interactive Dynamics of Cell Volume and Cell Death in Human Erythrocytes Exposed to α-Hemolysin from *Escherichia coli*

**DOI:** 10.3390/ijms23020872

**Published:** 2022-01-14

**Authors:** Nicolas A. Saffioti, Natalia Lauri, Lucia Cané, Rodolfo Gonzalez-Lebrero, Karina Alleva, Isabelle Mouro-Chanteloup, Mariano A. Ostuni, Vanesa Herlax, Pablo Julio Schwarzbaum

**Affiliations:** 1 Laboratorio de Biosensores Avanzados, Instituto de Nanosistemas, Universidad Nacional de San Martín, San Martín, Buenos Aires 1650, Argentina; nsaffioti@gmail.com; 2 Departamento de Química Biológica, Facultad de Farmacia y Bioquímica, Universidad de Buenos Aires, Junín 956, Buenos Aires 1113, Argentina; natti.lau@gmail.com (N.L.); lolo@qb.ffyb.uba.ar (R.G.-L.); pschwarzbaum@gmail.com (P.J.S.); 3 Instituto de Química y Físico-Química Biológicas “Prof. Alejandro C. Paladini”, UBA, CONICET, Facultad de Farmacia y Bioquímica, Junín 956, Buenos Aires 1113, Argentina; kalleva@ffyb.uba.ar; 4 Instituto de Investigaciones Bioquímicas de La Plata (INIBIOLP) CCT-La Plata, CONICET, Facultad de Ciencias Médicas, Universidad Nacional de La Plata, 60 y 120, La Plata, Buenos Aires 1900, Argentina; lucane21@hotmail.com (L.C.); herlax@hotmail.com (V.H.); 5 Université de Paris and Université des Antilles, INSERM, BIGR, F-75015 Paris, France; isabelle.mouro-chanteloup@inserm.fr

**Keywords:** purinergic, cell volume regulation, RTX toxins, aquaporins

## Abstract

α-hemolysin (HlyA) of *E. coli* binds irreversibly to human erythrocytes and induces cell swelling, ultimately leading to hemolysis. We characterized the mechanism involved in water transport induced by HlyA and analyzed how swelling and hemolysis might be coupled. Osmotic water permeability (P*_f_*) was assessed by stopped-flow light scattering. Preincubation with HlyA strongly reduced P*_f_* in control- and aquaporin 1-null red blood cells, although the relative P*_f_* decrease was similar in both cell types. The dynamics of cell volume and hemolysis on RBCs was assessed by electrical impedance, light dispersion and hemoglobin release. Results show that HlyA induced erythrocyte swelling, which is enhanced by purinergic signaling, and is coupled to osmotic hemolysis. We propose a mathematical model of HlyA activity where the kinetics of cell volume and hemolysis in human erythrocytes depend on the flux of osmolytes across the membrane, and on the maximum volume that these cells can tolerate. Our results provide new insights for understanding signaling and cytotoxicity mediated by HlyA in erythrocytes.

## 1. Introduction

α-hemolysin (HlyA) is an exotoxin secreted by uropathogenic strains of *E. coli* (UPEC) that causes lysis of several cell types, including red blood cells (RBCs) from humans and other mammalian species [1,2,3]. HlyA belongs to the repeats-in-toxin (RTX) family, a group of bacterial toxins sharing a glycine- and aspartate-rich motif repeated in tandem [4].

The severity of urinary tract infections correlates with the expression levels of HlyA in UPEC isolates, suggesting that HlyA is an important virulence factor [5,6,7]. Moreover, HlyA has been associated with complicated urinary tract infections, pyelonephritis and bacteremia in humans [5,8,9], whereas intravenous inoculation of the toxin in animal models enhanced lethal sepsis [10]. Although HlyA at high doses is highly lytic, relatively low toxin concentrations are thought to mimic the environment found during infections of the urinary tract, which are sometimes accompanied by discrete cell lysis and exfoliation of the bladder epithelial cells [11].

UPEC strains can translocate to the circulatory system, where they synthetize and release HlyA to the intravascular milieu. HlyA can then bind to and irreversibly insert into the RBCs cell membrane [12], inducing an increase of transmembrane ionic permeability of low selectivity [13]. It is a matter of discussion whether these changes are produced by HlyA generating a defined pore (as observed in the unrelated α-hemolysin from *Staphylococcus aureus* [14,15]) or by producing lesions in the cell membrane by a detergent-like mechanism [16]. Permeability changes seem complex, since the size of the pore or lesions was suggested to increase with time of toxin exposure, thus allowing a time-dependent incremental exchange of osmolytes [16]. It is also not clear whether HlyA is active as a monomer [17] or requires oligomerization inside the cell membrane to become active [18,19].

Regarding sublytic effects observed in RBCs, HlyA induced Ca^2+^ influx causing activation of the Ca^2+^ activated K^+^ channel (KCa3.1) and the transmembrane protein 16A (TMEM16A) (K^+^ and Cl^−^ channels, respectively) [20]. The resulting elevated cytosolic Ca^2+^ triggered eryptosis (the apoptosis of RBCs, see [21]), a process involving activation of calpains that proteolyze α and β-spectrins of the cytoskeleton [22], translocation of phosphatidylserine to the external layer of the plasma membrane [23], generation of ceramide [22] and concomitant decreases in RBCs deformability [23]. Although the acute effects of HlyA may induce cell shrinkage, the toxin was reported to trigger compensatory cell swelling. In RBCs, this is achieved by HlyA-dependent accumulation of extracellular ATP (ATPe), leading to activation of ionotropic purinergic P2X receptors, followed by activation of the influx of osmolytes and water [23,24].

In several studies, there has been focus on HlyA inducing changes in the membrane permeability to ions, which then leads to osmotic imbalance and osmotically determined changes of cell volume. However, HlyA may also affect cell volume by affecting water permeability in different ways.

In RBCs, although rapid changes in water transport are governed by aquaporin 1 (AQP1) [25], other mechanisms, such as AQP3 [26], the urea transporter B (UT-B) [27], and simple diffusion through the lipid membrane, can also mediate water transmembrane fluxes. In principle, about two-thirds of water transport occurs via AQP1, an abundant protein in human RBCs [27,28]. The importance of AQP1 in RBCs was confirmed using RBCs from the rare variants of the Colton-null phenotype (AQP1 null RBCs), which do not express AQP1 and, therefore, exhibit low water permeability [28]. Whether calpain activation by HlyA can directly affect AQP1, or any other member of the AQP family, is currently unknown. Alternatively, the toxin could affect water transport due to perturbation of the lipidic environment [29].

In this work, we analyzed, for the first time, the water transport pathways during cell volume increase by HlyA. Among the various osmotic and cytotoxic effects exerted by the toxin on RBCs, we also analyzed whether hemolytic activity can be explained based only on osmotic stress created by HlyA, or else other processes increasing cell fragility are involved.

We characterized changes in mean corpuscular volume (MCV) and water transport elicited by HlyA through electrical impedance, flow cytometry and stopped-flow side light scattering, whereas cell rupture was monitored by hemoglobin release and light dispersion. Based on our results, we propose a simplified mathematical model of HlyA activity that explains the experimental data. In this model, kinetics of cell volume and hemolysis of RBCs results from the net movement of osmolytes across the membrane induced by the toxin.

## 2. Results

Results of this study describe the dynamics of MCV, changes in osmotic permeability (P*_f_*), and lysis of HlyA-treated RBCs. We present experimental results, as well as a data-driven model, to account for the relationships between these processes. Given the importance of AQP1 for facilitating water transport in RBCs, key experiments were repeated using AQP1-deficient RBCs (denoted as _AQP1null_RBCs).

### 2.1. Pre-Lytic Volume Changes

**Figure 1:**
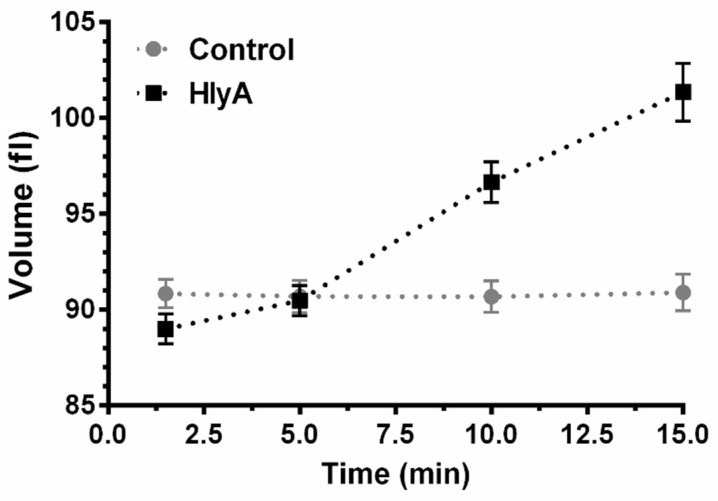
RBCs at 10% hematocrit were incubated in the absence of toxin (Control), or in the presence of 100 ng/mL HlyA (HlyA) at 37 °C during the times indicated in the figure. Then, MCV was measured by impedance cell cytometry, as indicated in Section 4.9. Points represent the mean values and error bars represent standard error of the mean (S.E.M.). (Control, N = 20; HlyA, N = 15).

To understand how HlyA produces volume changes in RBCs, cells were incubated in the absence and presence of the toxin, and MCV was measured as a function of time using impedance cell cytometry (Figure 1).

In the absence of HlyA (i.e., control), MCV was roughly constant. Exposure to the toxin led to a slight reduction in MCV after 1.5 min, followed by continuous non-linear swelling. 

Shape dynamics assessed by optical microscopy showed MCV changes to be accompanied by morphological transitions (see Supplementary Figure S1A and Supplementary Video S1). A detail of these morphological changes can be seen in scanning electron micrographs (Supplementary Figure S1B).

### 2.2. P_f_ in HlyA Treated RBCs

**Figure 2:**
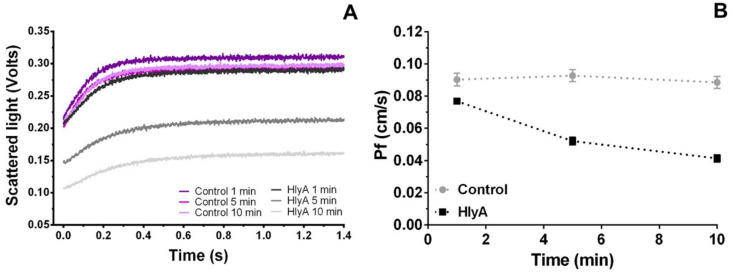
(**A**) SSC kinetics of HlyA pre-treated RBCs. RBCs suspensions at 10% hematocrit were treated with HlyA 100 ng/mL for 1, 5 and 10 min at 37 °C. After preincubation, cells were exposed to a hyperosmotic medium and the osmotic shrinkage of the cells was monitored by measuring the SSC signal in stopped-flow equipment as indicated in Section 4.7. Control experiments were performed in the absence of toxin. (**B**) Osmotic water permeability (P*_f_*) as a function of time of HlyA preincubation. Values of P*_f_* were calculated as detailed in Section 4.8 from SSC data. Data is represented as a function of the time during which cells were pre-exposed to 0 (control) or 100 ng/mL HlyA before stopped-flow experiments were performed. Values represent means ± S.E.M. (Control, N = 18; HlyA, N = 15).

The P*_f_* of HlyA-treated RBCs was measured to study the mechanism allowing MCV changes elicited by the toxin (Figure 1). Cells were suspended in isosmotic medium, and preincubated with 0 (control) or 100 ng/mL HlyA for different time periods, followed by exposure to a 455 mosM (mosm/kg H_2_O) hyperosmotic medium. This procedure created an osmotic transmembrane gradient of 140 mosM. Rapid volume changes were then estimated by monitoring the 90° side scatter light (SSC) using stopped-flow spectrophotometry (Figure 2A). Under the experimental conditions, the SSC signal was inversely proportional to MCV.

Preincubation of RBCs with HlyA elicited a decrease in P*_f_* (Figure 2B), indicating that the toxin reduces water transport through the RBCs membrane, thus causing a 50% P*_f_* reduction in 10 min. P*_f_* of RBCs was high (0.09 cm/s), so that upon a 140 mosM gradient the osmolarity of the cytoplasm was equilibrated in less than a second with the external osmolarity. In contrast, MCV changes induced by HlyA, shown in Figure 1, occurred in minutes. Therefore, P*_f_* is not the rate-limiting step controlling the nonlinear increase of MCV observed in Figure 1. 

### 2.3. Effect of Purinergic Signalling on P_f_

HlyA induces the release of intracellular ATP, leading to accumulation of ATPe [23,24]. Elevated ATPe induced by HlyA and other stimuli plays an important role in amplifying RBCs swelling by acting on ionotropic P2X purinergic channels [30,31]. 

**Figure 3:**
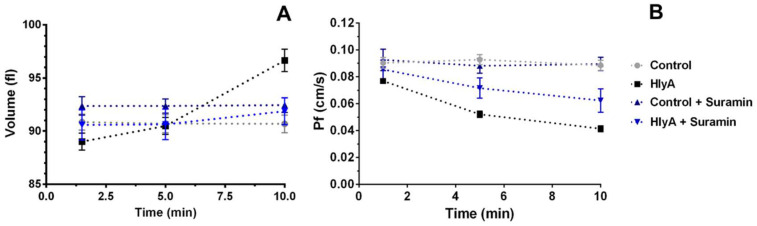
Effects of Suramin on MCV and P*_f_* changes induced by HlyA. RBCs suspensions at 10% hematocrit were used to determine MCV (**A**) and P*_f_* (**B**) after 1, 5 and 10 min of HlyA treatment (100 ng/mL). P*_f_* values were calculated, as indicated in Section 4.8, from stopped-flow experiments. Before HlyA addition, cells were pre-incubated in the absence or presence of suramin 100 μM for 10 min at 37 °C. Control experiments were run in the absence of toxin. Points in the plots represent means ± S.E.M. In (**A**) Control, N = 20; Control + Suramin and HlyA + Suramin, N = 5; HlyA, N = 15. In (**B**) Control, N = 18; Control + Suramin and HlyA + Suramin, N = 4; HlyA, N = 15.

Thus, MCV and P*_f_* of HlyA-treated RBCs were measured in the absence and presence of suramin, a generic antagonist of P2, and in the absence and presence of HlyA. Our results show that the addition of suramin delayed cell swelling induced by HlyA (Figure 3A), to the extent that MCV kinetics were roughly similar in the absence and presence of toxin.

When assessing P*_f_*, no effect was observed with suramin alone (Figure 3B), while treatment with both suramin and HlyA caused a 27% reduction after 10 min of toxin exposure. This relative decrease of P*_f_* was significant, but nevertheless weaker than the 49% reduction observed with HlyA in the absence of the purinergic blocker.

The kinetics of both MCV and P*_f_* as a function of toxin exposure time seem to be related, considering that P2 blockage induced a reduction of both swelling and P*_f_*. 

### 2.4. Effects of MCV on P_f_

Since swelling caused by HlyA, as observed in Figure 1, may be the primary cause for the observed changes in P*_f_*, we decided to run additional experiments with RBCs preincubated in different hypoosmotic media (to trigger swelling), in the absence of toxin. In this way, different initial MCV levels could be generated and measured. Subsequently, cells were exposed to hyperosmotic media of different osmolarities, to create a 140 mosM osmotic gradient (similarly to that used for P*_f_* measurements using HlyA-treated cells) between the intracellular and the extracellular compartments. Accordingly, for RBCs preincubated in 190, 215, 245 and 260 mosM media, hyperosmotic media of 346, 370, 400 and 415 mosM were used.

Having determined MCV and P*_f_* values for RBCs exposed to HlyA and hypo-osmotic media, it was then possible to plot P*_f_*-MCV relationships for RBCs pre-exposed to the different conditions*,* i.e., hypo-osmotic media, HlyA, and HlyA-suramin.

**Figure 4:**
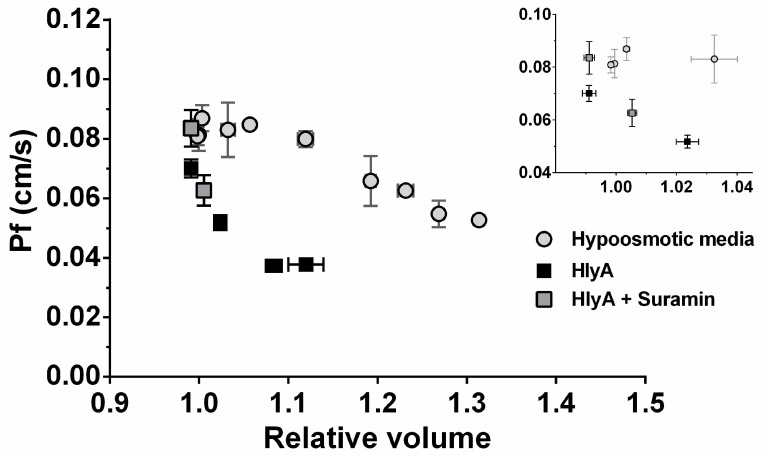
Changes in P*_f_* as a function of the relative MCV. Cells were incubated 1–10 min with 100 ng/mL HlyA in the presence of 100 μM suramin (gray square) or in its absence (black square), or were exposed to hypo-osmotic media (190–260 mosM, grey circles). After incubation, different aliquots of RBCs suspensions at 10% hematocrit were used to determine MCV, and to perform stopped-flow experiments, as described in Section 4.7. P*_f_* values were calculated as described in Section 4.8. The relative MCV was calculated by dividing the measured MCV by the MCV of control RBCs in isosmotic medium. For the preparation of this figure, the results were grouped in 8 groups based on the measured relative volume ranges (0.95–1, 1–1.05, 1.05–1.1, 1.11–1.15, 1.15–1.2, 1.2–1.25, 1.25–1.3, 1.3–1.35 relative volume). Then relative cell volumes and P*_f_* values within each range were averaged. Inset: Magnification of the main plot between 0.95–1.04 relative volume. Results are means ± S.E.M. (Hypo-osmotic media, N = 5; HlyA, N = 8, HlyA and Suramin, N = 4).

In Figure 4, MCV values were expressed relative to the isotonic control. For both HlyA- and hypo-osmolarity challenged RBCs, P*_f_* values decreased inversely with MCV, though the patterns were different for the two treatments. The main difference being that under HlyA treatment, unlike hypo-osmotic exposure, P*_f_* values decreased more steeply for small increments of MCV. These results demonstrate that cell swelling does affect P*_f_*. However, the different behavior profiles using HlyA vs. hypo-osmotic media showed that the HlyA-dependent effects are not entirely a consequence of its capacity to induce swelling. This can be clearly observed in the inset of Figure 4, showing that P*_f_* steeply decreased with HlyA, with no significant changes when using hypo-osmotic media.

Thus, HlyA affects P*_f_* by volume-dependent and volume-independent mechanisms.

### 2.5. Role of AQP1 in HlyA Activity

In RBCs, aquaporin 1 (AQP1) [25], aquaporin 3 (AQP3) [26] and the urea transporter type B (UT-B) [27] can facilitate water transmembrane transport. In addition, water can cross the RBCs membrane by simple diffusion. Among these mechanisms, AQP1 is the major water transporter. Therefore, reduced AQP1 activity may explain the observed decreases in P*_f_* in the presence of HlyA. To assess this possibility, we evaluated the effects of HlyA on P*_f_* of _AQP1null_RBCs [32].

Since _AQP1null_RBCs were thawed from frozen samples, it was necessary to perform control experiments using frozen samples from healthy individuals. Therefore, we worked with three types of cells: control RBCs (c-RBCs), control RBCs from frozen samples (cf-RBCs), and _AQP1null_RBCs. The results obtained from experiments using _AQP1null_RBCs were always compared with those using cf-RBCs, i.e., normal RBCs subjected to the same freeze and thaw treatment performed on _AQP1null_RBCs. Thus, any difference observed between cf-RBCs and _AQP1null_RBCs can be assigned to the absence of AQP1 on the latter.

**Figure 5:**
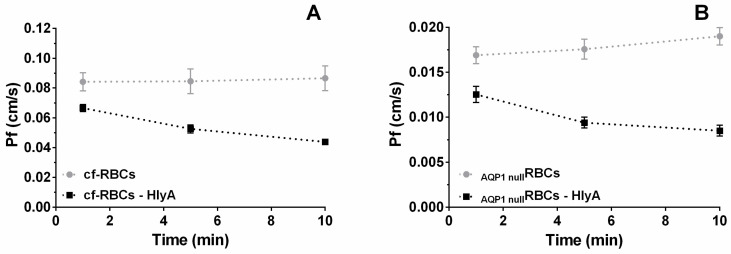
P*_f_* as a function of pre-incubation time with HlyA for control RBCs from frozen samples (cf-RBCs) or _AQP1null_RBCs. The plots show the P*_f_* obtained from cf-RBCs (normal RBCs expressing AQP1 that were subjected to the same freeze and thaw procedure as _AQP1null_RBCs) (**A**) or from _AQP1null_RBCs (**B**). Suspensions of RBCs at 10% of hematocrit were pre-treated with 100 ng/mL HlyA (black squares) or without toxin (gray circles) for 1, 5 or 10 min at 37 °C. Then, stopped-flow experiments were performed as indicated in Section 4.7. P*_f_* was calculated as indicated in Section 4.8. Control experiments were run in the absence of toxin (Control and HlyA conditions on both cf-RBCs and _AQP1null_RBCs, *n* = 3). Data are means ± S.E.M.

Exposure to HlyA in _AQP1null_RBCs induced a P*_f_* reduction from 0.02 to 0.009 cm/s after 10 min. This represents a 0.011 cm/s reduction of P*_f_*, a value about 5 times lower than that obtained using cf-RBCs (Figure 5A) or in c-RBCs (Figure 2B). Nevertheless, in both cell types, the relative decrease in P*_f_* induced by HlyA at 10 min amounted to approximately 50%.

**Figure 6:**
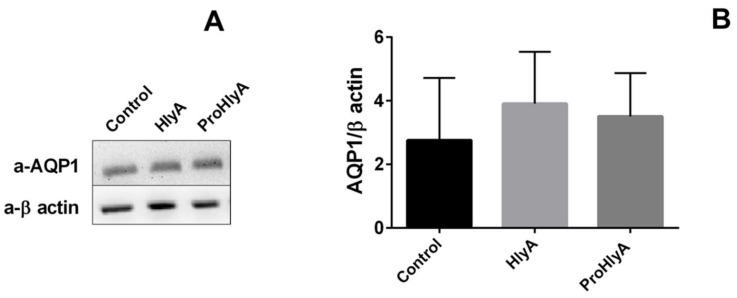
Western blot analysis of AQP1 in RBCs membranes. (**A**) RBCs suspensions at 10% hematocrit were treated with vehicle (Control), HlyA 100 ng/mL and ProHlyA 1 μg/mL at 37 °C for 10 min. The first row corresponds to the AQP1 band revealed with anti-AQP1 antibody. The second row corresponds to the anti-β actin antibody. (**B**) Densitometric analysis of the bands in A, expressed as the ratio between optical density of AQP1 and β-actin bands (N = 3).

The decrease in P*_f_* observed in Figure 5A could in principle be due to activation of intracellular proteases induced by HlyA [22] which would then proteolyze AQP1 and decrease AQP1 transport. Thus, we performed a Western blot experiment to study the integrity of the AQP1 in RBCs treated with HlyA. Densitometry analysis of AQP1 bands in Figure 6B indicated similar AQP1 protein levels for RBCs exposed to 0 (control) or 100 ng/mL HlyA. Incubation with the inactive toxin ProHlyA provided similar results. 

The fact that in HlyA-treated cells the relative decrease of P*_f_* is similar in RBCs and _AQP1null_RBCs, suggests that the toxin may affect other water transport mechanisms in addition to AQP1. 

#### Kinetics of Hemolysis, and Its Relationship to Changes in MCV and P*_f_*

To determine whether AQP1 is essential for MCV increase and hemolysis induced by HlyA, kinetics of MCV and hemolysis were evaluated in _AQP1null_RBCs vs. cf-RBCs. Hemolysis rates increased with toxin concentration, with T_50_ decreasing in a near exponential fashion. Although hemolysis rate was 44% lower in _AQP1null_RBCs (vs cf-RBCs), at relative high toxin concentrations, all RBCs samples were fully hemolyzed at the end of the incubation period.

**Figure 7:**
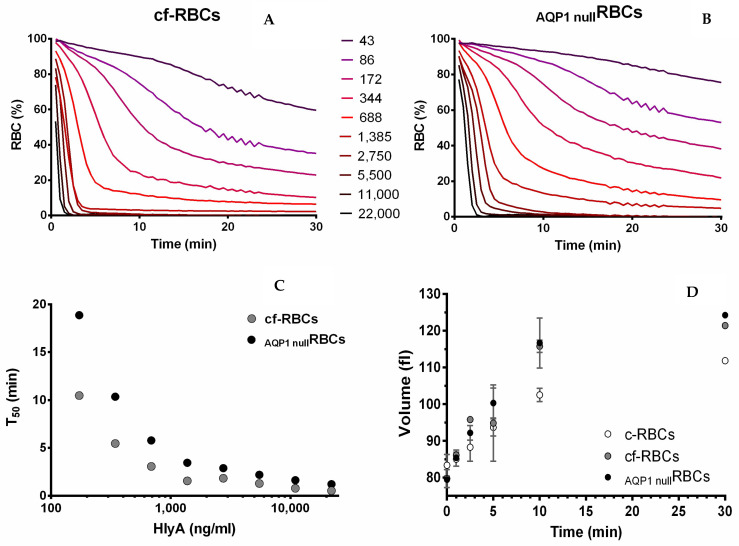
Hemolysis and volume kinetics in _AQP1null_RBCs and cf-RBCs. In panels (**A**,**B**), the proportion (%) of viable cells for control RBCs from frozen samples (cf-RBC, **A**) or _AQP1null_RBCs (**B**) is shown. Data were obtained as indicated in Section 4.6.2, using RBCs suspensions at 1% hematocrit. The concentration of HlyA for each curve is indicated between panels (**A**,**B**) in ng/mL. Panel (**C**). Data of panels (**A**,**B**) were used to calculate the necessary time for achieving 50% hemolysis (T_50_). A semi-logarithmic plot shows values of T_50_ vs. HlyA concentration. Panel (**D**). MCV as a function of incubation time with 100 ng/mL HlyA was measured for fresh RBCs (c-RBCs, white circles), control frozen RBCs (cf-RBCs, gray circles) and _AQP1null_RBCs (black circles). Panels (**A**–**C**) show data from a representative experiment. Results in (**D**) represent means ± S.E.M. (*n* = 3).

On the other hand, toxin-dependent swelling of _AQP1null_RBCs was similar to that of c-RBCs after incubation with HlyA (100 ng/mL) at several times (Figure 7D). Thus, the observed 75% lower values of P*_f_* of _AQP1null_RBCs did not affect HlyA effects on MCV. Moreover, the lower hemolysis rates of _AQP1null_RBCs could not be explained in terms of changes in MCV. 

### 2.6. How Cell Volume Is Linked to Hemolysis

HlyA hemolysis can, in principle, be explained by swelling until a maximal critical MCV (MCV_max_) is attained, after which lysis occurs. However, HlyA also affects both RBCs cytoskeleton and the cell membrane [22,33] which could make the cell more fragile and unable to tolerate large volume changes. To the extent that the HlyA-induced increase in cell fragility may facilitate hemolysis, the MCV_max_ should be smaller in the presence than in the absence of toxin. To test this hypothesis, we measured the kinetics of MCV by electrical impedance cell cytometry and hemolysis by hemoglobin release when cells are challenged by 40 ng/mL HlyA or by hypo-osmotic media.

**Figure 8:**
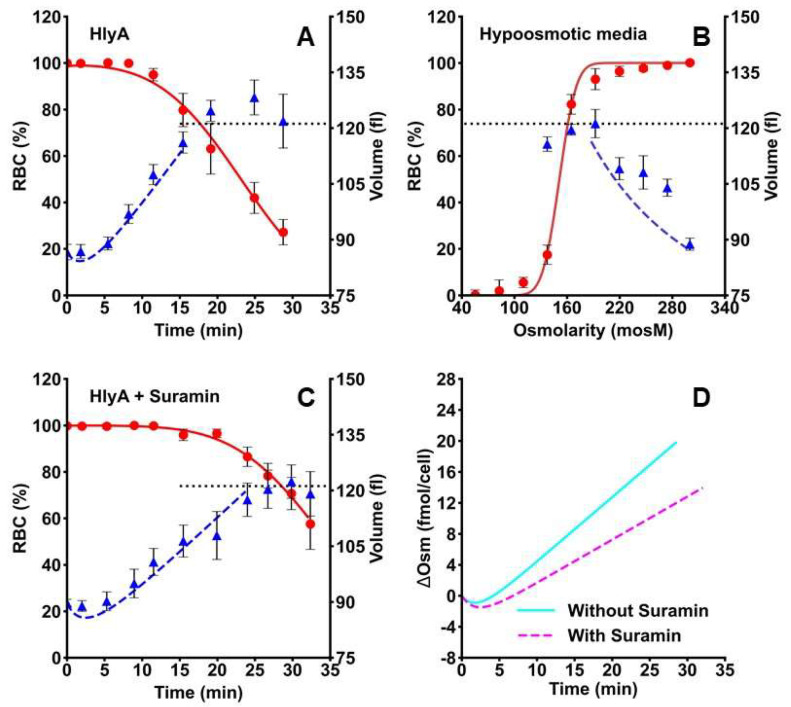
Effects of external osmolarity or HlyA on cell volume and hemolysis of RBCs. RBCs suspensions at 5% hematocrit were employed for experiments. Panel (**A**). Kinetics of MCV (blue triangles) and hemolysis (red circles) in the presence of 40 ng/mL of HlyA. Hemolysis was measured by the release of hemoglobin (as indicated in Section 4.6.1) and expressed as the proportion (RBC %) of viable RBCs. Volume was measured by electrical impedance cytometry (as indicated in Section 4.9) (N = 4). Panel (**B**). MCV (blue triangles) and hemolysis (red circles) as a function of media osmolarity (N = 3–6). Panel (**C**). Kinetics of MCV (blue triangles) and hemolysis (red circles) in the presence of 40 ng/mL HlyA plus 100 μM suramin. The black horizontal dotted line represents the MCV_max_ achieved in hypo-osmolar media. Values are means ± S.E.M. (N = 3–5). In panels (**A**,**C**), the dashed and continuous lines represent the predicted values of MCV and hemolysis respectively, obtained by fitting model 2 to experimental data (see Section 2.7). The dashed and continuous line in panel B represents the predicted values of MCV and hemolysis, respectively, obtained by fitting model 1 to experimental data (see Section 2.7). Panel (**D**). the plot shows the traces that represent values of ΔOsm vs. time predicted by model 2. The traces were obtained by fitting model 2 to data in Figure 8A,C.

Hemolysis kinetics showed an initial lag phase when steep swelling occurred (Figure 8A).

At approximately 11 min, MCV amounted to 105 fl when significant hemolysis started to be detected. At 20 min, average values of MCV stabilize near 120 fl, while the hemolysis speed was maximal. The MCV_max_ was 128 ± 5 fl at 25 min post-toxin exposure. 

To test whether this MCV_max_ is the maximum physically possible MCV for RBCs, we measured the MCV and hemolysis in different hypo-osmotic media in the absence of toxin. When plotting MCV as a function of media osmolarity, hemolysis can be detected at osmolarities lower than 200 mosM (Figure 8B). At about 137 mosM, more than 80% of RBCs were lysed, while the MCV_max_ of the remaining viable cells was 121 ± 4 fl. Therefore, in RBCs similar MCV_max_ values can be obtained in the absence or presence of HlyA.

To validate results of MCV kinetics, similar experiments using flow cytometry were performed. Forward light scatter (FSC) of RBCs suspensions was measured, where the signal is directly proportional to MCV. In agreement with results of Figure 8, the maximum value of FSC achieved in the presence of HlyA (isosmotic medium) was similar to that obtained in hypo-osmotic media (Supplementary Figure S3). 

To understand the role of ATPe and purinergic signaling in the hemolytic mechanism of HlyA, we assessed the kinetics of MCV and hemolysis after toxin addition in the presence of suramin (Figure 8C). At 40 ng/mL HlyA, changes in MCV and hemolysis were reduced, but not abolished by the presence of the P2 blocker, thus suggesting that ATP signaling is modulating, rather than mediating MCV increase and hemolysis elicited by HlyA. 

### 2.7. Mathematical Models Explaining HlyA-Dependent Kinetics of MCV and Hemolysis

To explain the dynamics of MCV and hemolysis induced by hypo-osmotic media or by HlyA, two simple models were built based on the work of Poole (40) (see Appendix A for further details). In both models, MCV changes and hemolysis are the consequence of an osmotic gradient across the plasma membrane (osm_grad_).

In Model 1, RBCs are challenged by hypo-osmotic media. In this model, MCV varies to counterbalance the osm_grad_, i.e., to make [Osm]_i_ = [Osm]_e_ and hemolysis increases as [Osm]_e_ reaches a critical value. Model 2 is different, because no external hypo-osmotic medium is imposed. In this case, RBCs are challenged by HlyA dissolved in isosmotic medium. Here, MCV and hemolysis depend on an osm_grad_ gradient created by the balance of osmolytes across the membrane (ΔOsm).

Model 1. Changes MCV and hemolysis on hypo-osmotic media

This model was employed to assess data of MCV and hemolysis when RBCs were exposed to hypo-osmotic media (Figure 8B). MCV changes vs. [Osm]_e_ were predicted by Equation (A2), which considers that MCV varies to equilibrate [Osm]_e_ and [Osm]_i_. Predictions of Equation (A2) followed the trend of experimental MCV data as a function of osmolarity until 200 mosM, after which, experimental data and the equation diverge (dashed blue line, Figure 8B). In particular, the equation does not agree with data once MCV_max_ is reached, because cells can no longer compensate the osmolarity change by swelling. At osmolarities lower than 200 mosM, hemolysis started to be detected. 

To analyze hemolysis as a function of [Osm]_e_, Equation (A5) was employed. This equation represents the % of RBCs that is lysed at any [Osm]_e_. By fitting Equation (A5) to data (red continuous line Figure 8B), the value of best fit of parameter μ was 150 ± 2 mosM. This parameter indicates the [Osm]_e_ at which 50% of RBCs were lysed, which means that RBCs can withstand, on average, an osm_grad_ of 150 mosM (i.e., [Osm]_i_ = 300 mosM vs. [Osm]_e_ = 150 mosM).

Model 2. HlyA-dependent changes in MCV and hemolysis

HlyA-dependent changes in MCV and hemolysis were calculated from a simple equation (Equation (A6)) describing the balance of osmolytes across the membrane (ΔOsm) vs. time in the presence of HlyA. Then, the values of MCV (Equation (A8)) and hemolysis (Equation (A9)) were calculated as a function of ΔOsm. Model 2 was fitted to MCV and hemolysis experimental data shown in Figure 8A,C. A good fit of the model to experimental data was observed, indicating that the dynamics of MCV and hemolysis can be explained solely on osm_grad_ induced by the toxin. 

The dynamics of ΔOsm predicted by model 2 after the addition of HlyA and suramin is shown in Figure 8D. In the absence of suramin, our model predicts that an osm_grad_ is generated rapidly because the osmolytes influx (mainly driven by Na^+^) overcomes the contribution of osmolytes efflux (mainly driven by K^+^). In the presence of suramin, P2 blockage decreased cation influx, thus resulting in slower osmolyte incorporation by RBCs (Figure 8D).

Results agree with those of Figure 3A, where MCV changes were inhibited in the presence of suramin.

**Figure 9:**
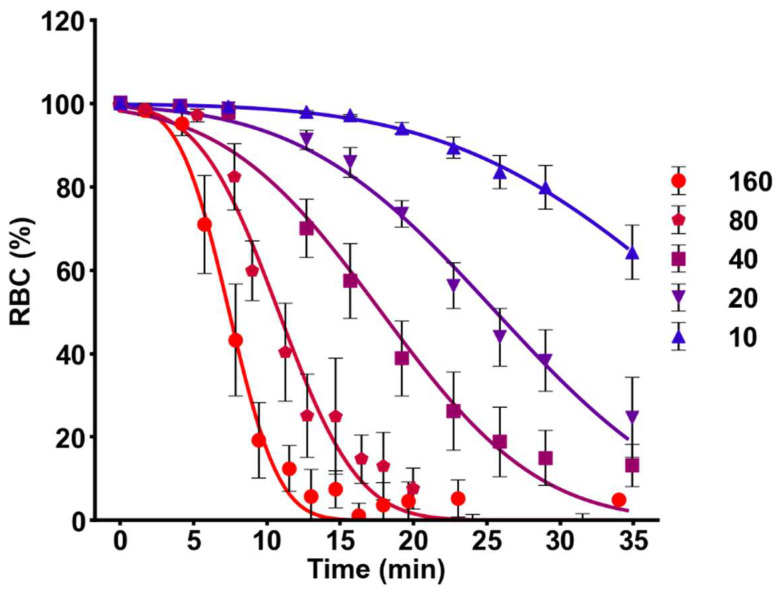
Fitting of time-dependent effect of HlyA on hemolysis. RBCs suspensions at 5% hematocrit were treated for different times with 5 HlyA concentrations (10–160 ng/mL). Data are expressed as the proportion of viable cells (RBC%) vs. time. The continuous lines show the fit of model 2 to experimental data at each HlyA concentration. Best fit values of the parameters are shown in Supplementary Table S1. Data represents means ± S.E.M. (N = 3–6).

Finally, to test if model 2 can explain HlyA activity, we measured the hemolysis kinetics by quantifying the release of hemoglobin at various toxin concentrations (10–160 ng/mL). The model obtained a good fit to experimental data (Figure 9), suggesting that HlyA-hemolysis can be described by changes in osm_grad_ over a wide range of toxin concentrations.

## 3. Discussion

Despite various studies describing the effects of HlyA on ion permeabilities and cell volume, the direct effects of the toxin on water permeability, and associated changes in cell volume and hemolysis, have not been assessed until now. Swelling is especially deleterious to RBCs which, unlike most other cells, lack cell volume regulatory mechanisms to counteract volume increase [34].

Exposure of RBCs to HlyA induced a significant decrease of P*_f_*. The fact that the response to the toxin depended on exposure time is consistent with the proposed mechanism of toxin action [16]. A similar P*_f_* reduction pattern was observed in control- and _AQP1null_RBCs, indicating that HlyA affects one or various water transport mechanisms, such as AQP3, UT-B, and/or simple diffusion across the membrane [23]. The observed lack of direct effects of HlyA on AQP1 is supported by Western blot results showing neither proteolysis nor loss of AQP1 in membranes of toxin-treated RBCs. Unlike the lack of effect on AQP1, HlyA was shown to activate calpains producing partial proteolysis of Band 3, another abundant membrane protein of RBCs [22].

Experiments comparing decreases in P*_f_* due to HlyA vs. exposure to hypo-osmotic media show that HlyA-dependent P*_f_* decrease was not entirely due to the capacity of the toxin to induce swelling, so that other signaling steps should be responsible for the observed reduction of water permeability. In this respect, previous reports showed that P2 receptor activation induced HlyA-dependent swelling of RBCs [23,24] and, this study shows that blocking P2 not only inhibits swelling, but also partially inhibited the strong P*_f_* inhibition induced by HlyA. 

On the other hand, AQP1 disruption did not change MCV kinetics in RBCs, in line with individuals of the rare Colton null phenotype not suffering clinical symptoms [28], probably because sufficient non-AQP1 water permeability is able to control cell volume under HlyA exposure. Thus, relative slow water movements in RBCs may be secondary to net fluxes of osmolytes driving the volumetric response. This ion-dependent induction of water flux was interpreted in terms of a data-driven mathematical model.

Cytotoxicity of HlyA is commonly believed to correlate with acute Ca^2+^ entry activating K^+^ efflux, followed by subsequent Na^+^ influx and water [20]. Since toxin effects are time dependent, the model predicted the rates of osmolyte transport across the membrane (ΔOsm) as a function of time, causing changes in MCV, while hemolysis occurs when ΔOsm values surpass the maximal number of osmolytes that an RBCs can physically incorporate before exploding. The model predicted that in HlyA-treated RBCs, early K^+^ efflux is partially compensated by Na^+^ influx, consistent with the observed slow initial phase of swelling. Later, net Na^+^ uptake is greatly accelerated, followed by osmotically obligated water influx, and swelling, leading to hemolysis.

When studying more closely the cell volume-hemolysis coupling, a lag phase of hemolysis was observed, while swelling was fast until cells achieved an MCV_max_ value before bursting. Using different hypo-osmotic media in the absence of toxin, a similar MCV_max_ was observed, suggesting that HlyA influence on cell volume and hemolysis relies mainly on osmotic effects. Nevertheless, under HlyA exposure a small population of cells hemolyzes before MCV_max_ is achieved, thus pointing to higher cell fragility under toxin exposure. 

Results of this study may help understand the contribution of HlyA to the virulence of UPEC strains, where toxin can alter different signaling cascades of the host. Although in vitro HlyA-dependent volume and hemolysis kinetics do not appear to depend on high P*_f_* mediated by AQP1, RBCs are mobile cells that encounter different environments as they travel through the circulation. Rapid water exchange mediated by AQP1 is assumed to protect RBCs from osmotic stress when these cells permeate the hypertonic renal medulla, a situation where HlyA may impair the capacity of RBCs to trigger an adaptive response. On the other hand, since AQP1 is very abundant in the proximal tubule kidney and contributes 10 times more to P*_f_* than UT-B [35], HlyA reduction of P*_f_* might affect water reabsorption in the kidney. Consistent with this idea, AQP1-deficient individuals, as well as AQP1_null_ mice, show an impaired ability to concentrate urine [35,36]. Therefore, to the extent that P2 blockers partially inhibit HlyA effects on P*_f_*, they may offer a novel protection mechanism of clinical value against toxin-induced osmotic stress, thus limiting HlyA function as a virulence factor.

In addition, the results may shed light on other exotoxins inducing similar osmotic and purinergic events as HlyA. In this respect, leukotoxin A is another RTX toxin, a virulence factor produced and released by the bacterium *Aggregatibacter actinomycetemcomitans*, which can cause localized aggressive periodontitis and endocarditis. Interestingly, compared to HlyA, leukotoxin A causes qualitatively similar changes in the volume of RBCs, similarly modulated by P2X activation [30], though effects on P*_f_* were not studied.

## 4. Methods

All reagents in this study were of analytical grade. Bovine serum albumin (BSA), suramin, sucrose, chloramphenicol, guanidinium hydrochloride, ethylene glycol-bis(2-aminoethylether)-N,N,N′,N′-tetraacetic acid (EGTA) and phenylmethanesulfonyl fluoride (PMSF) were purchased from Sigma (St. Louis, MO, USA).

### 4.1. Media Used

- *Isosmotic medium*: (in mM) 137 NaCl, 2.7 KCl, 2.5 Na_2_HPO_4_, 1.50 KH_2_PO_4_, 1.32 CaCl_2_, 1.91 MgSO_4_, 5 glucose, pH 7.4 at 25 °C, 300 mosM.
- *Hypo-osmotic medium*: isosmotic medium reduced in NaCl content to obtain 55, 82, 110, 137, 164, 192, 219, 247 and 274 mosM.
- *Tris-Chloride (TC) buffer*: (in mM) 20 Tris, 150 NaCl, pH 7.4 at 25 °C.

### 4.2. Isolation of RBCs

- *Fresh RBCs:* RBCs were obtained from healthy individuals by venipuncture. After blood collection, the sample was centrifuged at 900*× g* for 3 min and the plasma, the platelets and the leucocytes were removed and discarded. Isolated RBCs were resuspended and washed three times in isosmotic medium. Finally, packed RBCs were resuspended at the corresponding hematocrit. The procedure was approved by the Ethics Committee on Clinical Investigation of the School of Pharmacy and Biochemistry, University of Buenos Aires (Res RESCD-2020-281-E-UBA-DCT_FFYB).
- *Frozen RBCs:* Control RBCs (cf-RBCs) and RBCs from an individual lacking the major water channel aquaporin 1 (_AQP1null_RBCs) [27] were provided by the Centre National de Référence des Groupes Sanguins (Paris, France). Before the study, cryopreserved RBCs of both conditions were thawed and washed in isosmotic medium as described for fresh RBCs.

### 4.3. Toxin Purification

HlyA was purified from culture of the overproducing *Escherichia coli* strain WAM 1824 (*E. coli* JM15 strain transformed with pSF4000 containing *hlyCABD* operon). WAM 1824 was grown in Luria–Bertani medium at 37 °C in presence of 20 μg/mL chloramphenicol to late log phase and an OD_600nm_ of 0.8–1.0. Cells were pelleted and the supernatant was treated with 20% (*v*/*v*) cold aqueous ethanol at pH 4.5 (pI of HlyA) to precipitate and partially purify the toxin. Precipitated toxin was collected by centrifugation (14,500× *g* at 4 °C for 1 h, Sorvall centrifuge rotor SSA 34) and then resuspended in TC buffer supplemented with 6 M guanidinium hydrochloride. Protein content was determined by Bradford assay [37]. SDS-PAGE revealed a main band at 110 kDa corresponding to more than 90% of the total protein. The resulting proteins were stored at −70 °C in guanidinium hydrochloride.

### 4.4. RBCs Treatment

RBCs suspensions (1–10% hematocrit) were incubated in the presence of HlyA for the times indicated in each figure. Before each experiment, fresh toxin solutions were prepared from a stock solution. Controls were run in the absence of toxin.

For experiments using suramin, RBCs were pre-treated with 100 μM of this drug for 10 min at 37 °C before HlyA addition. 

For measurements in hypo-osmotic media, the RBCs were pre-incubated in different osmolarity media (55–280 mosM) for several minutes at the temperature the experiment was performed.

### 4.5. Microscopy

Videomicroscopy and scanning electron microscopy (SEM) were used to study cell morphology.

#### 4.5.1. Videomicroscopy

To determine real-time shape changes of viable cells, RBCs suspensions at 1% of hematocrit were exposed to vehicle or HlyA, mounted immediately on slides and observed by light microscopy (Biotraza LCD XSP-167SP). During 30 min, images were acquired using a coupled camera at 100× magnification. Experiments were run at 20 °C.

#### 4.5.2. Scanning Electron Microscopy

RBCs were treated with vehicle or HlyA and then fixed using 2.5% glutaraldehyde. Fixed RBCs were coated with graphite by thermal evaporation of charcoal (0.150 Torr at 50 Amp for 30 s) by means of a desk carbon accessory coater (Denton Vacuum, Moorestown, NJ, USA). Coated samples were placed in a holder and observed by SEM (JEOL model JSM- IT300-LV, Tokyo, Japan). The images were acquired with a secondary electron detector at 30 kV, 10–30 spot size and working distance of 10 mm under high vacuum.

### 4.6. Hemolysis Assays

The hemolysis of HlyA-treated RBCs was determined by two different methods:

#### 4.6.1. Quantification of Hemoglobin Release at 405 nm

Hemolysis was evaluated by measuring free hemoglobin released to the medium by lysed RBCs. A quantity of 5% *v*/*v* of RBCs suspension was treated with different HlyA concentrations at 37 °C for 40 min. During this period, aliquots of the RBCs suspension were taken at different times (the suspension was gently agitated each time to ensure a homogeneous distribution of the cells). The samples were centrifuged at 2680× *g* in an Eppendorf centrifuge for 30 s and free hemoglobin in the supernatant was quantified by light absorption at a wavelength of 405 nm [38] in a 96-well microtiter plate. In each experiment, the 405 nm absorption of the supernatant of a sample of RBCs in the absence of HlyA was measured (ABS_0_). To estimate the hemoglobin released at 100% hemolysis (ABS_100_), the absorbance of the supernatant of a RBCs suspension treated with 160 ng/mL of HlyA for 30 min at 37 °C was also measured. The proportion of remaining RBCs (RBC %) at each time was calculated by the following formula (where ABSs represents the absorbance of the sample):

(1) $$\mathrm{RBC}\left(\%\right)=100-\frac{100\left({\mathrm{ABS}}_{s}-{\mathrm{ABS}}_{0}\right)}{{\mathrm{ABS}}_{100}-{\mathrm{ABS}}_{0}}$$

#### 4.6.2. Measurement of Light Scattering at 595 nm

Hemolysis kinetics of _AQP1null_RBCs treated with HlyA was determined by light scattering at 595 nm. For this purpose, HlyA was serially diluted in isosmotic medium in a 96-well microtiter plate. Then, 100 μL of 2% hematocrit RBCs suspension was added. The plate was incubated at 25 °C and the absorbance at 595 nm was measured every 30 s during 30 min. ABS_0_ correspond to the absorbance at 595 nm of untreated RBCs and ABS_100_ to the 100% hemolysis sample as specified in Section 4.6.1. The RBC% was calculated using Equation (1).

### 4.7. Stopped-Flow Assay

To evaluate changes in P*_f_* induced by HlyA on RBCs variants, cells were treated with the toxin for fixed times and then the stopped-flow assay was performed.

Experiments were run using SFM 400 stopped-flow equipment (BioLogic, Grenoble, France). HlyA-treated and control RBCs were rapidly mixed with hyperosmotic medium. This hyperosmotic medium was prepared by the addition of 140 mosM sacarose to the same medium in which the cells were preincubated. Time courses of the changes in 90° scattered-light intensity (SSC) (λ_exc_ of 530 nm) of RBCs were measured to follow the osmotic shrinking of cells at 37 °C. At least 8 time-courses were averaged, and the following equation was fitted to the data:

(2) $$\mathrm{SSC}=A_{\infty }\left(1-e^{-\mathrm{kt}}\right)+A_{0}$$

SSC represents side-scattered light and t represents the time. A_∞_ and A_0_ represent the scattered light at time 0 and infinite respectively. The k parameter defines the speed of the signal change. A least squares procedure was applied to obtain the best values of the k, A_0_ and A_∞_ parameters.

### 4.8. P_f_ Calculation

P*_f_* (cm/s) was determined using the equation proposed by van Heeswijk and van Os [39]:

(3) $$P_{f}=\frac{V_{0}\;k}{{S\;V}_{w\;}C_{\mathrm{out}}}$$

where V_0_ is the initial volume of RBC, S is the cell area, V_w_ is the molar volume of water (18 cm^3^/ mol), k is the parameter obtained in Section 4.7 and C_out_ is the total concentration of extracellular solutes. C_out_ was controlled in every experiment using an osmometer (Vapro 5520, Wescor Inc., Logan, UT, USA). 

### 4.9. MCV Determinations

MCV measurements were performed using the automated cell analyzers MS4e (Melet Schloesing, Osny, France) and Casy (Schärfe System, Reutlingen, Germany) based on the Coulter counting principle by measuring changes in electrical impedance. For these determinations RBCs hematocrit was 10%. In the case of the MS4e, the equipment was purged with the hypo-osmotic media corresponding to the measurement to ensure that all dilutions and measurements were performed in the same medium. For assays in the presence of HlyA, measurements were performed in isosmotic media at different times after toxin addition. The volume histograms obtained from the MS4e cell analyzer were processed to express cell volume in femtoliter units as specified in Supplementary Figure S2.

### 4.10. RBCs Forward Light Scattering (FSC)

The light scattering of individual RBCs was measured by Accuri Plus 6 (BD, Franklin Lakes, NJ, USA). For measurements in different hypo-osmotic media, 5% hematocrit of RBCs suspensions were pre-incubated in each medium for 30 min at 37 °C. HlyA-treated RBCs experiments were performed in isosmotic media at different times after toxin addition at 37 °C.

### 4.11. Western Blot of AQP1

RBCs at 10% hematocrit were incubated in the presence of 100 ng/mL HlyA, 1 μg/mL ProHlyA or without toxin for 10 min at 37 °C. After treatment, cells were diluted with a buffer containing Tris 15 mM, EDTA 1 mM and PMSF 1 mM, pH 7.65 (membrane purification buffer) to induce cell lysis. Then, the samples were centrifuged for 15 min at 16,000 rpm, at 4 °C, the supernatant discarded and the pellet containing the RBCs plasma membranes resuspended in membrane purification buffer. Between 6 and 7 centrifugations were performed to remove all soluble proteins. Purified RBCs membranes were prepared for a 16% SDS-PAGE. Samples were resuspended in sampling buffer in the presence of 2% SDS. After electrophoresis, the gel was transferred to PVDF membranes which were blocked with 3% skim milk in TBS buffer (10 mM Tris-HCl, 150 mM NaCl, pH 7.4) at room temperature for 1.5 h. Then, they were incubated with a solution containing a polyclonal rabbit anti-AQP1 antibody (Alpha Diagnostic) (1:1000), washed with TBS buffer, and finally reacted with peroxidase-conjugated anti-rabbit antibody (Jackson) (1:30,000) in TBS buffer with 3% skimmed milk at room temperature for 1.5 h. Finally, the membranes were incubated with a peroxidase substrate solution (Immobilon Western, Millipore) for the detection of horseradish peroxidase-conjugate antibodies on the membrane. Images were obtained using ChemiDoc MP Imaging System (Bio-rad, Hercules, CA, USA).

### 4.12. Model Fit

The models of hemolysis and MCV changes elicited by HlyA were prepared as indicated in Appendix A and fitted to experimental data. The code was written in Python, employing pandas, scipy, matplotlib and numpy libraries. Details of the procedure can be found in Appendix A.

### 4.13. Statistics

In each figure, the number of independent experiments used to estimate the mean and standard error of the mean is indicated with the letter “N”. In the Figure 5 and Figure 7, the experiments were performed using _AQP1null_RBCs from the same donor, therefore, the number of independent assays is indicated with the letter “n”.

## Data Availability

Experimental data of the results within this work are available at the following site: https://doi.org/10.6084/m9.figshare.17978210.

## Supplementary Materials

The following are available online at https://www.mdpi.com/article/10.3390/ijms23020872/s1.

## Appendix A

The models presented here were fitted to experimental data of Figure 8A–C.

In the models, changes in MCV and hemolysis are the consequence of an osmotic gradient across the plasma membrane (osm_grad_). This gradient can be calculated by the difference between extracellular ([Osm]_e_) and intracellular ([Osm]_i_) osmolarities using the following equation:

(A1) $${\mathrm{osm}}_{\mathrm{grad}}={\left[\mathrm{Osm}\right]}_{i}-{\left[\mathrm{Osm}\right]}_{e}$$

**Model 1. MCV and hemolysis of RBCs exposed to hypo-osmolar media**

The MCV varies to counterbalance the osm_grad_, i.e., to make [Osm]_i_=[Osm]_e_. This can be represented by the following equation:

(A2) $$\mathrm{MCV}=\frac{{\left[\mathrm{Osm}\right]}_{i}}{{\left[\mathrm{Osm}\right]}_{e}}\left(V_{0}\left(1-f_{h}\right)\right)+V_{0}f_{h}$$

V_0_ is the MCV at isosmolar medium (300 mosM) and f_h_ is the fraction of MCV occupied by hemoglobin and other proteins (volume not occupied by water) which was taken from the work of Freedman and Hoffman (f_h_ = 0.35) [40]. The value of [Osm]_i_ is 300 mosM (which corresponds to MCV in isosmolar conditions), but it is recalculated when MCV changes due to the osmotic activity of hemoglobin [40]. The osmotic activity coefficient of hemoglobin was calculated using the equation proposed by Freedman and Hoffman:

(A3) $$\Phi_{\mathrm{Hb}}=1+0.0645\left[\mathrm{Hb}\right]+0.0258{\left[\mathrm{Hb}\right]}^{2}$$

where Φ_Hb_ is the osmotic activity coefficient of hemoglobin and [Hb] is the hemoglobin concentration inside the RBCs. The value of [Hb] at V_0_ was considered 7.5 mM [40]. At each MCV, the value of [Osm]_i_ was recalculated using the following equation:

(A4) $${\left[\mathrm{Osm}\right]}_{i}=\left[\mathrm{Hb}\right]{*\Phi }_{\mathrm{Hb}}+{\left[\mathrm{Osm}\right]}_{\mathrm{notHb}}$$

where [Osm]_notHb_ is the contribution of all osmolytes except for hemoglobin and its value was 278 mosM at V_0_.

Results of Figure 8B show that predictions from Equation (A2) follow the trend of MCV data as a function of [Osm]_e_ until 200 mosM, an osmolarity where experimental data and the equation diverge. Predictions of Equation (A2) do not agree with data once MCV_max_ is reached. This is because the cell can no longer compensate the osmolarity change by swelling.

To explain hemolysis at different [Osm]_e_, the maximal osm_grad_ that the RBCs population can tolerate was considered normally distributed. This means that 50% of all RBCs will lyse at a given osm_grad_ value called osm_50_. Also, there is a standard deviation of osm_grad_ (osm_dev_) whose value indicates how many RBCs lyse near osm_50._ A small osm_dev_ indicates that most RBCs lyse near osm_50_, while a big osm_dev_ indicates that RBCs lysis occurs over a wide range of osm_grad_. In hypo-osmotic media, osm_grad_ will depend on [Osm]_e_, as indicated in Equation (A1). Therefore, it could be predicted that the proportion of lysed RBCs as a function of [Osm]_e_ will be described by:

(A5) $$\mathrm{RBC}\%=\frac{100}{2}\left[1+\mathrm{erf}\left(\frac{{\left[\mathrm{Osm}\right]}_{e}-\mu }{{\mathrm{osm}}_{\mathrm{dev}}\sqrt{2}}\right)\right]$$

“Erf” refers to the error function, μ represents the [Osm]_e_ at which 50% of RBCs are lysed (when osm_grad_ = osm_50_) and osm_dev_ represents the standard deviation of the probability distribution. Equation (A5) was fitted to data of hemolysis vs. [Osm]_e_ and it is represented as a continuous red line in Figure 8B. The obtained best values of μ and osm_dev_ were 150 ± 2 mosM and 17 ± 2 mosM, respectively.

**Model 2. Changes in MCV and hemolysis of RBCs induced by HlyA**

MCV and hemolysis dynamics in the presence of HlyA dissolved in isosmotic medium are also a consequence of osm_grad_. However, in this case, osm_grad_ is not controlled by [Osm]_e_, but generated by toxin-induced osmolyte flux across the membrane, consequently producing cell volume changes and hemolysis in RBCs. The net balance of osmolytes across the membrane after RBCs enter in contact with HlyA is called ΔOsm. The osmolarity changes in HlyA-treated RBCs are driven by the balance between net K^+^ efflux and net Na^+^ influx.

The value of ΔOsm was calculated from the following equation:

(A6) $$\Delta \mathrm{Osm}=\mathrm{At}-B\left(1-e^{-k_{1}t}\right)$$

where parameter A accounts for the speed at which the RBCs incorporate osmolytes because of the inward Na^+^ flux. Parameter B contains information about the number of osmolytes lost by K^+^ efflux, and k_1_ is a kinetic constant that determines how fast the K^+^ contribution reach its equilibrium value.

ΔOsm is expressed in fmol/cell, but can be easily transformed in osm_grad_ by the following equation:

(A7) $${\mathrm{osm}}_{\mathrm{grad}}=\frac{\Delta \mathrm{Osm}}{V_{0}\left(1-f_{h}\right)}$$

The MCV changes and hemolysis in the presence of HlyA were calculated from ΔOsm. Therefore, to explain the MCV changes in terms of ΔOsm, the following equation was employed:

(A8) $$\mathrm{MCV}=\frac{\frac{\left[\Delta \mathrm{Osm}\right]}{V_{0}\left(1-f_{h}\right)}+{\left[\mathrm{Osm}\right]}_{\mathrm{notHb}}+\left[\mathrm{Hb}\right]{*\Phi }_{\mathrm{Hb}}}{{\left[\mathrm{Osm}\right]}_{e}}\left(V_{0}\left(1-f_{h}\right)\right)+V_{0}f_{h}$$

Equation (A8) is similar to Equation (A2), except that [Osm]_e_ is constant (cells were incubated in isosmotic medium) and [Osm]_i_ is equal to the sum of 3 terms: the contribution of all osmolytes with the exception of Hb ([Osm]_notHb_), the contribution of Hb osmotic activity ([Hb]*Φ_Hb_) and the osm_grad_ created by the toxin (calculated from ΔOsm). To explain the hemolysis kinetics in terms of ΔOsm we employed the following equation:

(A9) $$\mathrm{RBC}\%=\frac{100}{2}\left(1+\left[\mathrm{erf}\left(\frac{\mu_{\Delta \mathrm{osm}}-\Delta \mathrm{Osm}}{\sigma_{\Delta \mathrm{osm}}\sqrt{2}}\right)\right]\right)$$

“Erf” refers to the error function, μ_Δosm_ represents the number of incorporated osmolytes per cell that elicits the lysis of 50% of RBCs. σ_Δosm_ represents the standard deviation of the probability distribution (such as osm_dev_ in Equation (A5)).

For fitting Equation (A8) to experimental data, the value of μ_Δosm_ was set to 8.95 fmol osmolytes/cell. This value was calculated considering an MCV of 90 fl, an [Osm]_i_ of 300 osmM, and a fraction of 0.35 of the cytoplasm not occupied by water. Using these numbers, the number of osmolytes in the RBCs cytoplasm in isosmotic conditions is 17.55 fmol/cell. In Figure 8B, we calculated that half of the RBCs population is lysed at [Osm]_e_ = 150 osmM, which corresponds to an osm_grad_ of 50% of [Osm]_i_. This means that, on average, RBCs can withstand up to a 50% difference in the osmolarity across its membrane. Therefore, we considered that the RBCs could withstand up to a 50% increase in the cytoplasm osmolarity, which is achieved when ΔOsm = 8.95 fmol osmolytes/cell*,* i.e., when the cells have incorporated 8.95 fmol osmolytes as a consequence of toxin activity.

**Table A1:** Parameters obtained from model 2 fitting to data in Figure 8A,C. The best fit value of k_1_ was 0.5 min^−1^. However, the indetermination of the parameter (0.65 min^−1^) does not allow k_1_ to be expressed as the value ± standard error. Model fitting indicates that the value of k_1_ should be between 0 and 1.15 min^−1^.

Parameter	40 ng/mL HlyA without Suramin	40 ng/mL HlyA with Suramin
A (fmol/(cell*min))	0.92 ± 0.06	0.61 ± 0.05
σ_Δosm_ (fmol/cell)	5.0 ± 0.6	4.3 ± 0.7
B (fmol/cell)	4.3 ± 1.1	
k_1_ (min^−1^)	<1.15	
