# Supplementary material for: Interactive Dynamics of Cell Volume and Cell Death in Human Erythrocytes Exposed to α-Hemolysin from Escherichia coli
Source: Int J Mol Sci. 2022 Jan 14;23(2):872. doi: 10.3390/ijms23020872 (PMC8778525; doi:10.3390/ijms23020872)
Supplement: Supplementary file 1 [file ijms-23-00872-s001.zip › ijms-1538225-supplementary.pdf]

Interactive Dynamics of Cell Volume and Cell Death in Human Erythrocytes Exposed to α-Hemolysin from *Escherichia coli*

Nicolas A. Saffioti [1,2,†], Natalia Lauri [2,3,†], Lucia Cané [4], Rodolfo Gonzalez-Lebrero [2,3], Karina Alleva [3], Isabelle Mouro-Chanteloup [5], Mariano A. Ostuni [5,*], Vanesa Herlax [4] and Pablo Julio Schwarzbaum [2,3]

Supplementary material 1.

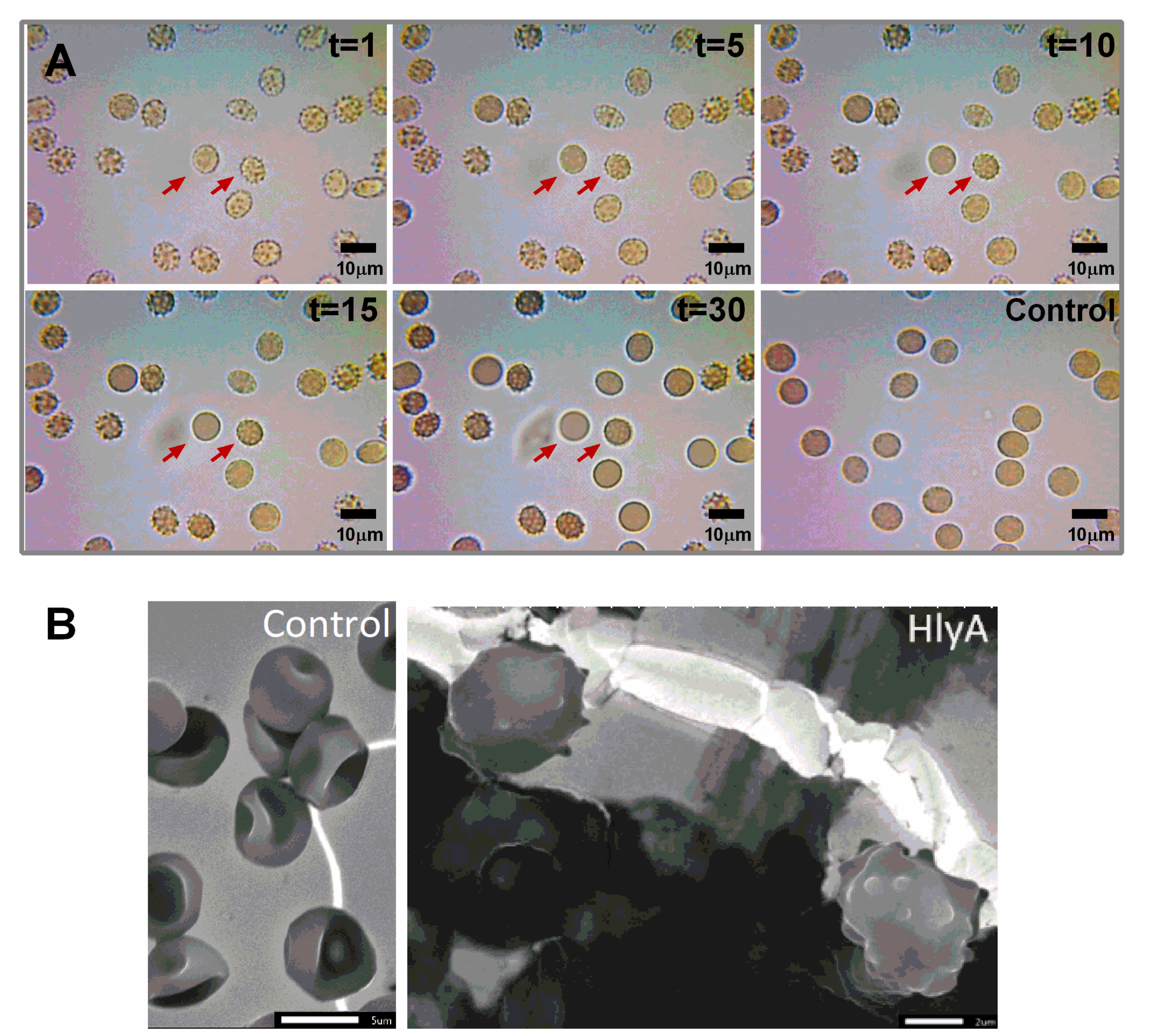

Figure S1. (A) Live cell imaging of HlyA treated RBCs. Light microscopy images of RBCs 1% hematocrit exposed to vehicle (Control) or HlyA 40 ng/ml at 20 °C for 1, 5, 10, 15 and 30 min (indicated on the top right of each picture). **(B) Surface Electron Microscopy (SEM) micrographs of HlyA treated RBCs.** SEM images of RBCs exposed to vehicle (Control; scale=5 µm) or HlyA 66 ng/ml (scale=2 µm). Representative images are shown (N=3).

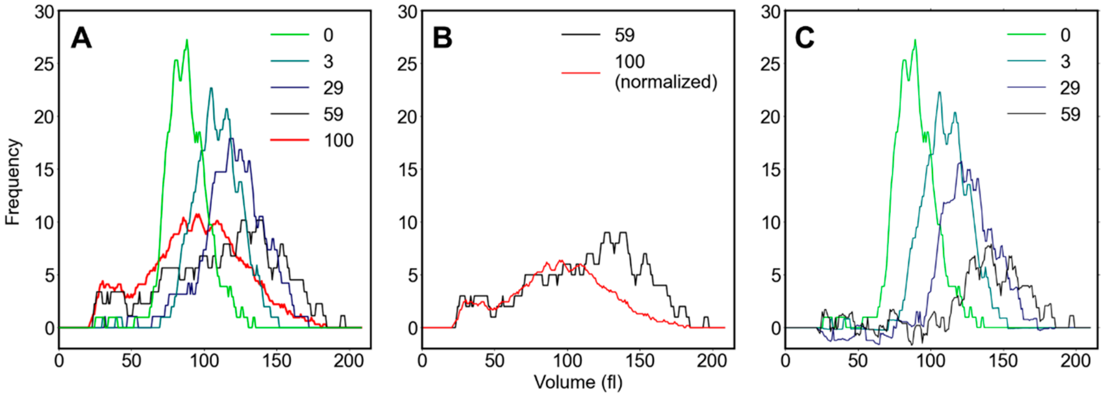

Figure S2. Correction of histograms for hemolysis. In panel A, the plot shows the cell volume histograms obtained at different times after exposure to RBCs suspensions. The histograms shifted towards the right due to cell volume increase. When hemolysis was significant, some events were detected at low volumes, probably due to formation of RBCs ghosts and/or cell debris. Hemolysis for each sample (%) is indicated on the top right.

To eliminate the interference of cell debris and RBCs ghosts in measurements, the histograms were corrected using the following formula:

$$Histogram_{corrected}(x) = Histogram_{raw}(x) - Histogram_{100\%}(x) * hemolysis \tag{S1}$$

At each interval of the histogram (x), the contribution of RBCs ghosts in the raw histogram ($Histogram_{raw}$) was estimated by multiplying the value of the histogram of 100% hemolysis ($\mathrm{Histogram}_{100\%}$) by the proportion of hemolysis in the sample. The RBCs ghost and cell debris contribution to the raw histogram was subtracted to calculate the corrected histogram for each sample that accounts only for viable RBCs.

In panel B, the plot shows the crude histogram from a sample with 59% hemolysis (black), and the calculated contribution of RBCs ghosts and cell debris (red). The red curve was calculated by multiplying each frequency value of the histogram of a sample with 100% hemolysis by a factor of 0.59.

The panel C shows the histograms plotted in panel A after the correction using Supplementary Eq. 1.

After the histogram correction, some frequency values displayed a low negative value (between -1 and 0) because of random variations in the data. For calculations of MCV using the corrected histograms, negative values were not considered.

* The histograms of 100% hemolysis were obtained by incubation of RBCs samples with 160 ng/ml of HlyA for 30 min. This incubation elicits a complete hemolysis of the sample. For each individual experiment, 3 different samples of 100% hemolysis were measured and averaged for histogram correction.

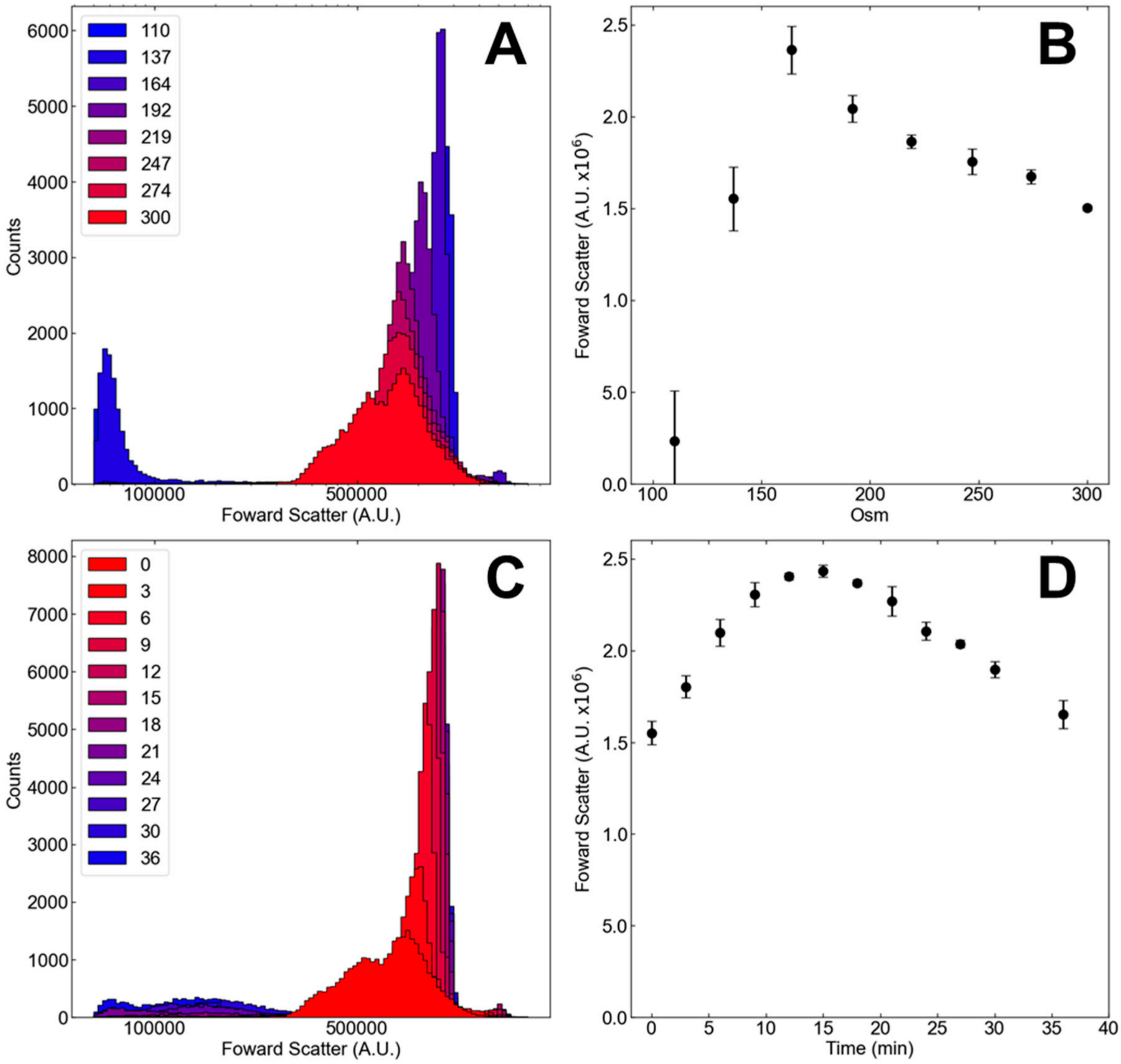

Figure S3. **Changes in MCV followed by flow cytometry forward scattering (FSC)**. In panel A the plot shows FSC histograms at different media osmolarity in the absence of HlyA. Each histogram was obtained at a different osmolarity indicated in the inset in mosM. As osmolarity decreases, the histograms shift to the right indicating an increase in MCV. However, note that a peak appears at the left of the plot when the osmolarity is lower than 164 mosM. We attributed this to RBCs ghosts and cell debris. The averaged FSC value from two independent experiments is plotted in panel B; error bars indicate the standard deviation between measurements. In panel C, the plot shows the histogram of FSC after the addition of 40 ng/ml of HlyA dissolved in isosmotic medium. In this case, the histograms shift towards the right as the time increases. After 15 min, RBCs ghosts and cell debris are detected in the left region of the histograms. The averaged FSC value from two independent experiments is plotted in panel D; error bars indicate the standard deviation between measurements. Note that the maximum value of FSC achieved in panels B and D is the same (2.4 x 10^6 A.U.). This indicates that the maximum MCV (MCV_{max}) is the same in both experiments: at low osmolarities in the absence of HlyA (Panels A and B) or in the presence of HlyA at isosmotic medium (Panels C and D).

Table S1. Best fit values of the parameters from model 2 fitted to experimental data of Figure 9.

HlyA (ng/ml)	σ (fosm/cell)	A (fosm/(cell*min))
160	4.3 ± 0.2	2.290 ± 0.005
80	4.8 ± 0.2	1.570 ± 0.002
40	5.7 ± 0.5	0.956 ± 0.002
20	4.9 ± 0.1	0.664 ± 0.001
10	4.060 ± 0.001	0.426 ± 0.001
